# Intensive Longitudinal Data Collection Using Microinteraction Ecological Momentary Assessment: Pilot and Preliminary Results

**DOI:** 10.2196/32772

**Published:** 2022-02-09

**Authors:** Aditya Ponnada, Shirlene Wang, Daniel Chu, Bridgette Do, Genevieve Dunton, Stephen Intille

**Affiliations:** 1 Khoury College of Computer Sciences Northeastern University Boston, MA United States; 2 Bouve College of Health Sciences Northeastern University Boston, MA United States; 3 Department of Population and Public Health Sciences Keck School of Medicine University of Southern California Los Angeles, CA United States

**Keywords:** intensive longitudinal data, ecological momentary assessment, experience sampling, microinteractions, smartwatch, health behavior research, mobile phone

## Abstract

**Background:**

Ecological momentary assessment (EMA) uses mobile technology to enable in situ self-report data collection on behaviors and states. In a typical EMA study, participants are prompted several times a day to answer sets of multiple-choice questions. Although the repeated nature of EMA reduces recall bias, it may induce participation burden. There is a need to explore complementary approaches to collecting in situ self-report data that are less burdensome yet provide comprehensive information on an individual’s behaviors and states. A new approach, microinteraction EMA (μEMA), restricts EMA items to *single, cognitively simple questions* answered on a smartwatch with single-tap assessments using a quick, glanceable microinteraction. However, the viability of using μEMA to capture behaviors and states in a large-scale longitudinal study has not yet been demonstrated.

**Objective:**

This paper describes the μEMA protocol currently used in the Temporal Influences on Movement & Exercise (TIME) Study conducted with young adults, the interface of the μEMA app used to gather self-report responses on a smartwatch, qualitative feedback from participants after a pilot study of the μEMA app, changes made to the main TIME Study μEMA protocol and app based on the pilot feedback, and preliminary μEMA results from a subset of active participants in the TIME Study.

**Methods:**

The TIME Study involves data collection on behaviors and states from 246 individuals; measurements include passive sensing from a smartwatch and smartphone and intensive smartphone-based hourly EMA, with 4-day EMA bursts every 2 weeks. Every day, participants also answer a nightly EMA survey. On non–EMA burst days, participants answer μEMA questions on the smartwatch, assessing momentary states such as physical activity, sedentary behavior, and affect. At the end of the study, participants describe their experience with EMA and μEMA in a semistructured interview. A pilot study was used to test and refine the μEMA protocol before the main study.

**Results:**

Changes made to the μEMA study protocol based on pilot feedback included adjusting the *single*-*question* selection method and smartwatch vibrotactile prompting. We also added sensor-triggered questions for physical activity and sedentary behavior. As of June 2021, a total of 81 participants had completed at least 6 months of data collection in the main study. For 662,397 μEMA questions delivered, the compliance rate was 67.6% (SD 24.4%) and the completion rate was 79% (SD 22.2%).

**Conclusions:**

The TIME Study provides opportunities to explore a novel approach for collecting *temporally dense* intensive longitudinal self-report data in a sustainable manner. Data suggest that μEMA may be valuable for understanding behaviors and states at the individual level, thus possibly supporting future longitudinal interventions that require within-day, temporally dense self-report data as people go about their lives.

## Introduction

### Background

Mobile technologies create new opportunities to design personalized health interventions in 2 broad ways. First, mobile technologies such as smartphones and smartwatches can be used to improve the assessment of behavioral, psychological, and contextual states, reducing reliance on known-to-be-problematic retrospective recall surveys that may poorly capture within-day variations [1-3]. Second, real-time measures gathered using mobile technologies can be used to design just-in-time adaptive interventions [4,5] that are sensitive to everyday changes in behaviors and states. State or behavior assessment and personalized interventions both require computational models that can represent the interrelationships between different behaviors and states unique to individuals. Such models could be created by using (1) a hypothetico-deductive approach to study relationships between predictors and outcomes of interest based on prior knowledge [6,7], (2) data-driven discovery from a large number of constructs measured in intensive longitudinal data (ILD) studies [8,9], or (3) an appropriate combination of both [10,11]. Creating models of behavior that capture relationships between behavior and context that can happen quickly and many times in a day requires methods for sustainable data gathering on, and modeling of, within-day changes of variables (eg, physical activity [PA], sleep, sedentary behavior [SB], and affect) [12,13].

Ideally, health-related constructs could be measured using passive sensors from easy-to-wear devices, without requiring any end-user effort other than wearing the devices and charging them. However, *we currently need self-report* *input* to measure many subjective experiences (eg, perceived pain, hunger, and fatigue) that play an important role in predicting health behavior changes [14]. A popular practice for obtaining self-report data on mobile devices is using experience sampling methods [15], also known as ecological momentary assessment (EMA) [16], to gather participant perspectives on in situ experiences that sensors cannot measure directly. As smartphones have become ubiquitous, EMA has become affordable to deploy with most adult populations [17].

In the most common EMA protocols used to collect ILD, participants’ smartphones prompt with audio or vibration several times a day, each time presenting a set of questions that may take a few seconds or minutes to answer, depending upon the number and complexity of the questions. Typically, questions are multiple choice, but sometimes they involve open-ended responses or sliding scales. The sampling frequency in EMA varies widely based on study goals, as does potential burden. Burden results from the annoyance of each EMA interruption and the time and mental effort required to answer the questions presented at the prompt [18,19]. To minimize response burden, researchers often reduce the number of questions in each question set, the complexity of the questions, the frequency of prompting, or all three, thereby limiting the scope and temporal density of the behaviors and states measured in an ILD study. Although intensive EMA protocols can be sustained for short-term studies (eg, 7-30 days), burden will accumulate in ILD collection studies that last several months or years [20]. This accumulated burden, if not managed, could prevent the conducting of large-scale longitudinal studies or interventions that rely on frequent self-report input.

Although compliance-contingent compensation is a common practice to boost or maintain EMA response rates, it can be expensive for large-scale longitudinal studies involving thousands of participants. Beyond observational studies, future personalized health interventions that may require self-report of participant states and contexts must balance information gathering with burden to ensure that long-term engagement does not wane. For instance, an intervention to reduce stress and increase PA may need to query users regularly about their perceived stress and physical exertion (among other subjective experiences) to adjust the intervention in real time. For such an intervention to be sustainable, the system may have to gather these data frequently at the rate at which stress and activity can change—that is, many times throughout the day—while keeping end-user burden manageable. For such an intervention to be affordable, financially compensating participants to motivate a reasonable response rate is not a viable long-term option. In fact, compliance-contingent compensation may result in poor data quality if the participants are only motivated by money [21]. Thus, new strategies are needed for acquiring temporally dense self-report on multiple constructs with sustainable burden, even for studies or interventions deployed for months or years.

A strategy for acquiring longitudinal self-report data is using microinteraction EMA (μEMA or micro-EMA) [22]. *Microinteractions* are actions lasting for 3-4 seconds (eg, checking the time on a watch or turning on a lamp); microinteractions are so short lived that they can be completed without disrupting ongoing activity [23]. µEMA is a type of EMA where all prompts are for *single questions* with *single-tap answers* (eg, *Nervous right now?* with answers *Yes*, *Sort of*, or *No*) [22]. Unlike EMA, where questions are prompted in a set, in µEMA a device prompts only a single question per interruption and there is a guarantee that the question can be answered with a microinteraction [23]. Because of this microinteraction guarantee, µEMA may enable self-report data collection at a much higher frequency than EMA. This at-a-glance response property is achieved in 2 ways. First, µEMA is deployed on wearable devices such as smartwatches that permit quick access to question content; unlike mobile phones, the questions can be seen with a flip of the wrist without additional time required to access a mobile phone that may be in a bag or out of sight [24,25]. Prior studies have demonstrated that smartwatches are more suitable for glanceable microinteractions (eg, checking notifications) than smartphones [26,27]. Second, μEMA questions are intended to be cognitively simple to answer (eg, *Feeling stressed?*—*Yes*, *Sort of*, or *No*) and with a limited answer set that fits on a small smartwatch screen and therefore does not require a scrolling interface. Prior work shows that reducing the number of answers (eg, from a 5-point Likert scale to a 3-point ordinal or nominal scale) improves response time without necessarily changing the perceived item difficulty [28]. The benefit of leveraging microinteractions is also supported by 2 laws in user interface (UI) design. First, Hick’s law posits that the more options there are to choose from, the longer the response time on the UI will be [29,30]. Second, Fitt’s law shows that the navigation time between 2 targets on a UI is directly proportional to the distance between the targets and inversely proportional to the size of the target [31,32]. However, the downside of restricting all prompted interactions to cognitively simple, glanceable, single-question microinteractions is that information obtained from a single µEMA question is more limited than what can be obtained from unconstrained EMA questions with multiple answer options (eg, *In the past one hour, how stressed did you feel?—Extremely*, *Quite a bit*, *Moderately*, *A little*, or *Not at all*). Presenting such EMA questions on a smartwatch screen would make smartwatch interaction cumbersome, requiring either a font size that would be too difficult for most people to read, especially for those who need reading glasses, or requiring scrolling. Furthermore, using self-report to capture feelings aggregated over longer time windows (eg, in the past hour or over the past day) introduces recall burden [18,33] and cognitive complexity. Therefore, EMA questions nearly always require adjustment to achieve the microinteraction property we seek.

Prior work has used wearable devices such as smartwatches and heads-up displays (eg, Google Glass) to deploy EMA question sets with the goal of making EMA easier. However, typically, such work has directly adopted surveys (with back-to-back questions) from mobile phone–based EMA surveys that require users to engage in prolonged interactions on the devices (eg, sliding or scrolling) [34-37] and thus do not result in microinteractions. Converting an EMA survey with multiple questions with multiple answers per question to a small smartwatch display has indeed required additional scrolling to make the questions and answers readable, thereby likely slowing down*—*not speeding up*—*answer selection and resulting in the smartwatch interaction becoming more burdensome than answering the same surveys on the mobile phone.

In 2 prior 4-week between-subject pilot studies, we found that despite experiencing approximately 4-6 times more interruption when using µEMA instead of mobile phone–based EMA, participants using µEMA on a smartwatch had significantly higher compliance, reported lower perceived burden, and answered with faster response times than participants when measuring the same constructs using EMA [22,38]. Prior work also demonstrates that for a single construct measured at high frequency, µEMA can yield good criterion validity when compared against a research-grade passive sensor (eg, in the domain of PA measurement) [39]. Table 1 summarizes key differences between the mobile phone–based EMA and smartwatch-based µEMA in a prior study [22] where both EMA and µEMA were used to measure positive and negative affect (using the Positive and Negative Affect Schedule [40] over a period of 4 weeks [41]).

With µEMA, a user is guaranteed that the worst-case interaction required to answer a prompt will always be a single, glance-and-tap microinteraction; thus, it is nearly as easy to answer a microinteraction prompt as it is to manually dismiss it (by swiping on the screen) or ignore it. If researchers develop simple questions appropriate for microinteractions that provide valuable information about constructs of interest, µEMA may support ILD studies that gather dense, within-day information on behavior. So far, researchers have used µEMA to measure only 1-2 constructs per study, such as hyperarousal [42], stress [43], and subjective comfort [44]; µEMA has also been used with situated displays in home settings [45,46]. µEMA is well suited for constructs that may require frequent self-report, such as chronic pain assessment [47]. µEMA can be assessed at such a high temporal density (ie, 4 times per hour) that it can also measure multiple constructs in a single day while maintaining reasonable temporal density of each construct.

Despite the promise of μEMA, much remains to be explored to determine the viability of the technique. In this paper, we present the μEMA protocol for the Temporal Influences on Movement & Exercise (TIME) Study. The TIME Study has a primary protocol that uses EMA, which is presented elsewhere (Wang, S, unpublished data, January 2022). Here, we present the protocol for a secondary, exploratory study within the TIME Study on the viability of using μEMA in an ILD study. The TIME μEMA substudy is the first large-scale ILD study (duration: 1 year) examining how μEMA might be used to measure multiple health behaviors such as PA, SB, and sleep along with time-varying subjective states (eg, stress, fatigue, and happiness). First, we describe the overall goal of the TIME μEMA substudy. Next, we detail the µEMA app we designed for the study, followed by reporting the qualitative feedback received from our pilot study participants. Subsequently, we present details of changes made to the protocol in response to pilot testing. Finally, we present preliminary compliance results from a subset of participants in the main study (81/246, 32.9%).

**Table 1:** Differences between mobile phone–based ecological momentary assessment (EMA) and smartwatch-based micro-EMA (μEMA; from prior work [22]).

	Mobile phone–based EMA	Smartwatch-based μEMA
Prompts per day	≤7	≤48
Prompting frequency	Once in 2 hours	Four times an hour
Number of questions per prompt	≤6	Only 1 question
Example of question framing	Over the past hour, how stressed did you feel?	Feeling stressed?
Number of response options per question	≥4 (including *choose all that apply* responses)	≤3 (eg, *Yes*, *Sort of*, and *No*)
Interruption burden	High (must access mobile phone, unlock it, and then start to answer)	Low (smartwatch always accessible with glance)
Response burden	High, because of multiple questions with more answer options	Low, because of only 1 question with limited answer options; aim is cognitive simplicity

### TIME μEMA Substudy Objectives

The goal of the TIME Study is to examine daily and within-day microtemporal processes (eg, feeling stressed, increased workload, being with family, and being at home) that may influence PA, SB, and sleep in young adults [48]. EMA is being deployed to capture reflective processes, reactive processes, internal factors, and external factors and assess how they affect health behavior adoption and maintenance (Wang, S, unpublished data, January 2022). Reflective processes are those that are slow and require careful deliberation (eg, intention to engage in healthy behaviors) [49]. In contrast, reactive processes are quick and automatic (eg, being on a regular routine) [49]. Internal factors are physiological and emotional sensations that originate internally (eg, positive and negative affect, pain, and fatigue). External factors are social, situational, and physical settings or events that originate externally to the individual (eg, an increase in workload or meeting a friend). An exploratory aim of the TIME Study is to assess the feasibility of using μEMA to gather similar health behavior data from participants. The fundamental difference between using EMA and μEMA for ILD can be summarized as follows. Traditional EMA is a method that interrupts less often than μEMA but asks for substantially more information with each interruption. In contrast, μEMA is a method that interrupts more often than EMA but asks only 1 simple question with each interruption. The TIME μEMA substudy will permit exploration of what we can learn about health behavior from small amounts of information gathered at high frequency using µEMA; the TIME substudy data will allow investigators to study the following research questions:

1. How sustainable is μEMA for ILD studies compared with mobile phone–based EMA?
  Interrupting participants 4 times more often with a single, simple μEMA question could be perceived as far more burdensome than interrupting less often with a longer, more complex EMA survey. Can participants in a full-year ILD collection study sustain μEMA with high compliance compared with EMA?
2. How do contextual factors influence μEMA compliance?
  Prior EMA literature has shown that contextual factors such as time of day, day of week, and location influence whether the participant is able to complete the EMA survey [50]. Behavior, such as being active, could also influence response rates. The TIME μEMA substudy will permit exploration of the contextual factors that may influence μEMA compliance and comparison of such effects between μEMA and EMA.
3. Can intermittent μEMA questions provide information on an individual’s overall behavior and state?
  Sustaining intensive mobile phone–based EMA longitudinally may not be realistic, requiring long temporal gaps between measurements and more retrospective recall. The TIME μEMA substudy will permit exploration of whether using μEMA *between* EMA burst periods could provide information on the diurnal patterns of behaviors and states not captured in the EMA bursts alone.
4. How can we use μEMA data *together with* data acquired from the passive sensors and EMA to design predictive models of health behavior change and maintenance?
  Each instance of μEMA self-report provides information on a single variable at a particular time, but μEMA prompts will be temporally dense, measuring different behaviors and states throughout the day. The TIME μEMA substudy will permit exploration of whether the amount and quality of μEMA data acquired in the TIME μEMA substudy is sufficient to build ideographic predictive models of behavior, either alone or in combination with EMA and passive sensing data.

In the remainder of the paper, we describe the protocol for the TIME μEMA substudy, from which ILD will be derived that will support such work.

### μEMA Design Overview

Deploying μEMA requires designing a specific smartwatch-based graphical UI and the question scheduling strategy.

### Custom μEMA App Interface

**Figure 1:**
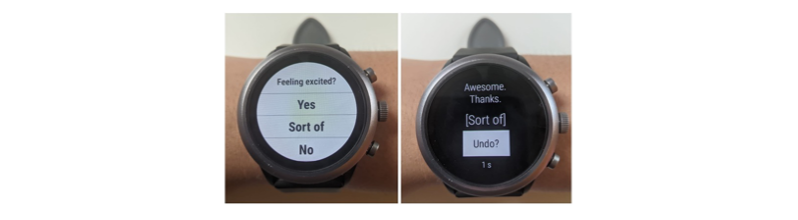
(Left) A microinteraction ecological momentary assessment question screen with 3 answer options, which will display for 20 seconds or until an answer is selected. (Right) A thank-you screen with the Undo? button indicating that the user selected the Sort of answer; the screen displays for 3 seconds with a countdown timer before it disappears.

We developed a custom μEMA app (called the TIME app or *app*) that runs on a smartwatch (Android Wear OS version 2.0) with a linked app on the paired smartphone (Android OS version ≥7.0). The Android OS was chosen because it provides flexibility in gathering raw sensor data from both smartphone and smartwatch continuously in the background. The apps work together to present μEMA questions on the smartwatch, collect and process passive sensor data, and transfer data to a research server each day. Each μEMA prompt presents only 1 question at a time that can be answered in a glance and a tap (a microinteraction). Example questions are *Feeling stressed?* and *Feeling productive today?* with three answer options: *Yes*, *Sort of*, and *No*. The smartwatch prompts the participant with a vibration pattern lasting for 3 seconds. The question screen displays at the start of the vibration (Figure 1). The brightness of the screen is determined automatically by the smartwatch based on ambient brightness or the smartwatch settings selected by the participant.

The question stays visible on the screen for 20 seconds, after which the question disappears and a missed response is recorded. If participants answer a question, they are presented with an acknowledgment screen with a short thank-you message and an *Undo?* button (Figure 1). The thank-you messages on the acknowledgment screen are selected from >250 unique variations designed to reduce repetition and boost engagement—they are all pithy, and some are quirky. The undo option is available for 3 seconds. If participants tap on the undo button, they are taken back to the question screen and they can then change their answer within 20 seconds. If they do not undo within 3 seconds, the app records the final answer and the question disappears. Participants have only 1 chance to undo an answer. If the app is dismissed (by swiping right on the screen) when the original question appears, it is recorded as *never started*, and if the app is dismissed on the undo screen, it is recorded as *completed then dismissed*. Similarly, if the user does not answer the question again after selecting the undo button, then the prompt status is recorded as *partially completed.*

### μEMA Prompt Scheduling

**Figure 2:**
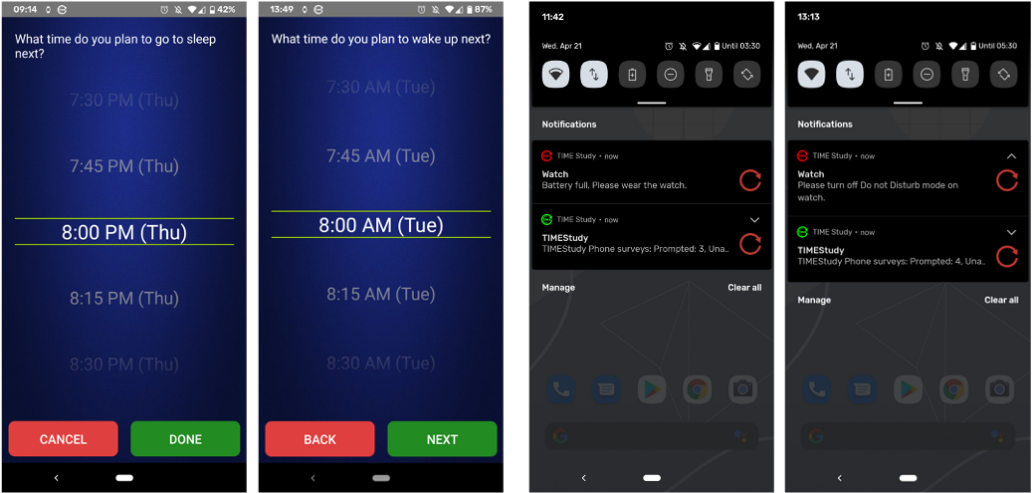
(Left) A mobile phone survey asking for prospective sleep and wake times. These times are used to adjust the prompt scheduling. (Right) Example notifications: (1) a reminder to wear the smartwatch prompted when it is still on the charger and is 100% charged and (2) a reminder to turn off the do-not-disturb mode on the smartwatch, which appears if that mode is active for >60 minutes consecutively.

μEMA prompts occur during waking hours, including when the smartwatch is being charged or is off the wrist. Participants self-report their upcoming sleep time and the next day’s wake time using EMA on their smartphone (Figure 2); this information is used to compute the waking hours and is sent to the smartwatch in real time. If participants do not respond to the sleep- and wake-time questions on the smartphone or if the smartphone cannot connect to the smartwatch for any reason, the smartwatch uses the previous day’s wake and sleep times as the default waking schedule to determine the μEMA prompting schedule. The purpose of the sleep-wake questions is to predetermine the prompting schedule for the day. Once answered, these sleep- and wake-time questions are presented again after 10 hours, in case participants want to change their schedule. Moreover, participants can use the app to manually change the sleep-wake times at any time of the day. μEMA prompting starts 15 minutes after each day’s wake time and ends 15 minutes before that day’s sleep time. For instance, if the participant plans to sleep at 11 PM and wake up the next day at 6 AM, then μEMA prompting will stop at 10:45 PM and resume at 6:15 AM the next day. Thus, days with longer waking hours will result in more μEMA prompts than days with shorter waking hours. μEMA questions are prompted 4 times an hour at random, with at least eight minutes guaranteed between 2 consecutive prompts using the following formula. The app generates this schedule for the 24-hour period using the following equations and then only prompts during waking hours.

*P_n_* = 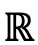 ∈ [0, *MaxTimeAvailable*_n_) + 8 + *P_n_*_–1_

*MaxTimeAvailable*_n_ = 55 – ((4 – n + 1) × 8) – *P_n_*_–1_

First, for each hour (eg, 8 AM to 9 AM), the app computes the maximum remaining time (*MaxTimeAvailable_n_*) in minutes to schedule a prompt (*P_n_*, where *n=1,...4,* P*_0_*=0) by subtracting the minimum required time gap (ie, 8-minute gap per prompt) and the time elapsed since the previous prompt within that hour (*P_n-1_*). Then, the app selects a random time from the *MaxTimeAvailable_n_* and adds the 8-minute gap and the prompt time of the previous prompt (*P_n_*_-1_) to determine the current prompt time (*P_n_*). The μEMA smartwatch app respects the settings on the smartwatch; therefore, prompting is paused when a participant turns on the do-not-disturb (DND) mode on the smartwatch. However, if the smartwatch is in DND mode for >60 consecutive minutes, participants receive a notification on the mobile phone to disable the DND mode on the smartwatch so that prompting can be resumed without further data loss (Figure 2). If the smartwatch is off the wrist, including when it is being charged, μEMA prompting continues normally.

Participants receive a notification on the mobile phone and smartwatch to (1) connect the smartwatch if it is disconnected from the mobile phone (through Bluetooth) for >60 consecutive minutes, (2) wear the smartwatch if the system detects that the smartwatch is fully charged but on the charger, (3) charge the smartwatch when the smartwatch battery reaches ≤15% capacity (Figure 2), and (4) update the software when the mobile phone or smartwatch software requires an update. Mobile phone and smartwatch notifications disappear as soon as the problem that was flagged is resolved. In addition, the mobile phone app presents a persistent notification indicating that the TIME app is running in the background collecting data and highlighting the countdown of when the next burst period on the mobile phone will start.

### System Components

The system has three components: mobile phone, smartwatch, and a remote server. The mobile phone prompts users with EMA surveys and end-of-day surveys. In addition, the mobile phone passively collects sensor data such as acceleration, location, and mobile phone use (eg, number of apps used). The details of mobile phone–related functionality are beyond the scope of this paper and are reported elsewhere (Wang, S, unpublished data, January 2022). The smartwatch prompts μEMA questions and passively collects raw accelerometer data (described in the next section). The data from the smartwatch are sent to the mobile phone either (1) once every 4 hours or (2) whenever the smartwatch is on the charger. The mobile phone then encrypts the data and sends it to a remote server daily. When the smartwatch or mobile phone are not transferring data automatically as expected, they each provide an option that permits participants to force the data transfer to the mobile phone; this functionality is used to resolve data transfer problems working remotely with the research staff.

## Methods

### Pilot Study

With the μEMA system described in the previous sections, we conducted a pilot study with 15 participants who answered μEMA questions for up to 3 consecutive weeks.

### Pilot Study Participants

Participants for the pilot study were recruited by means of flyers posted on university campuses in the Los Angeles metropolitan area and through targeted ads on Facebook. Participants were eligible to participate if they (1) were aged 18-24 years, (2) owned an Android smartphone with OS version ≥6.0, (3) were fluent in English, 4) were not planning to change their smartphone in the next 1 month, (4) were willing and able to wear a smartwatch and answer questions on their smartphone for a period of 1 month, and (5) were currently engaged in recommended levels of PA or intending to do so in the next 12 months.

### Pilot Study Design

The research staff met with the eligible participants in person (in 2019) and installed the TIME app on their smartphones. The app prompted participants with EMA surveys in 2 measurement burst periods. Each burst period lasted 4 days (called burst days). If participants completed at least eight EMA surveys per day in the first burst period, they were loaned a Fossil Gen 4 smartwatch (Fossil Group, Inc) and instructed on how to install the TIME smartwatch app. For the remaining 3 weeks, participants also collected data on the smartwatch. On the nonburst days, the smartwatch prompted with μEMA questions about a myriad of behaviors, including positive and negative affect, location, and PA. With each prompt, the smartwatch TIME app selected a question at random and presented it on the screen. In addition, questions about momentary PA, SB, and location (ie, *Physically active now?*, *Sedentary right now?*, and *At home right now?* with answer options *Yes*, *Sort* *of*, and *No*) were also selected randomly throughout the day. Thus, it was possible for a question to repeat consecutively when sampled at random. The goal of the pilot study is to assess the app’s performance, improve the study protocol, and fine-tune question wording and the overall user experience. At the end of the pilot study, participants took part in a structured interview about their experience of answering questions on the smartphone and smartwatch.

### Pilot Study μEMA Response Behavior

A total of 15 participants completed 1 month of data collection (3 weeks with μEMA prompting on the smartwatch). With 15,120 μEMA questions delivered, the μEMA response rate was 76.4% (SD 22.3%), with a mean μEMA response time of 4.3 (SD 1.0) seconds.

### Pilot Study Qualitative Feedback

At the end of the 1-month pilot study, participants completed a structured interview lasting for approximately 30 minutes to enable us to gather feedback on how to improve the user experience with the TIME app and to adjust the main study protocol accordingly. When asked about positive experiences with μEMA, participants highlighted the ease of access of the smartwatch and the simplicity of answering just 1 question. A participant described the experience as follows:

Honestly, it was the easiest to answer surveys on the watch because it’s right on your wrist and it vibrates [and] there is a survey, I answer the question and I am done...The watch questions are actually less burdensome than the phone questions. Watch questions are pretty convenient because you can just touch [tap] on the watch and you’re done basically.

F, aged 24 years

However, several participants felt that the μEMA question did not stay available for long enough. A participant stated as follows:

I like how it was really simple, it wasn’t like a series of questions. They were just “yes” or “no.” What made it difficult was the timing...it seems like it only lasts for five seconds and it disappears. Sometimes I may be doing something like resting and then survey comes up. Then I have to stick my arm out of the blanket to answer but then it goes away before I could.

F, aged 22 years

Initially, the prompt only remained active for 15 seconds, which we later updated during the pilot to 20 seconds. Participants generally noticed the smartwatch vibration on their wrists with a delay, despite a relatively aggressive vibration pattern. In fact, pilot participants described the μEMA vibration pattern (lasting for 11 seconds, intensifying progressively) as “too loud*.”* Although the smartwatch vibration creates a quiet buzzing sound when on the arm, if the smartwatch is sitting on a surface (eg, charging), the resulting surface vibration can make a surprisingly loud sound that is difficult to ignore.

Participants reported difficulty answering μEMA questions when both hands were occupied, especially while driving. However, they were instructed to only answer μEMA questions when it was safe to do so, and they were given the specific example of driving as a situation in which they should ignore the prompts. Participants also reported difficulty answering μEMA questions while doing other cognitively demanding activities such as writing. In fact, a participant mentioned missing prompts while being in an examination room, stating as follows:

Sometimes if I was taking a quiz or test in class, I didn’t want to be caught using my watch, so I didn’t touch [ie, answer] it.

M, aged 19 years

Participants found the ability to undo their responses useful, especially when there were accidental taps on the smartwatch. A participant stated as follows:

Yes [Undo] was useful because sometimes on the watch...maybe because of the clothes I’m wearing...but [if] an option would be chosen so I’d be able to go “undo” and choose the right answer.

F, aged 24 years

Participants also found the *Sort of* answer option helpful in instances when they were not sure of their experience at the moment and would have had difficulty entering a more limited *Yes* or *No* response. A participant stated as follows:

“*Sort of*” *made me think about my answer a little more rather than an outright yes or no, which is how I kind of determine how I’m going to answer yes or no...if you’re feeling such and such, [then] I do like the “sort of” option because you’re not feeling fully something. And you are not “not” feeling it completely either. So [“Sort of”] is a pretty easy option for people who are feeling like in the middle.* [F, aged 24 years]

In response to such observations with the goal of achieving cognitive simplicity to support microinteraction, we designed all the questions to have a *middle* answer such as *sort of*.

In the pilot study, each question presented was randomly sampled from the pool of questions. This resulted in questions either being overrepresented or repeated too consecutively for some individuals, adding monotony, which some participants noted. A participant stated as follows:

I think there were questions based on time or place. Questions like how was your day?...It would ask multiple times a day. I don’t know if it was intentional, but it feels like I’m answering the same question over and over again. Same thing for places. It would ask me multiple times “is this your home?” or something like this.

M, aged 18 years

### Changes to the Main Study Protocol

#### Overview

Table 2 summarizes the changes made to the μEMA app and protocol based on the pilot participants’ feedback. In summary, there were 4 major changes to the main protocol based on the data and participant feedback in the pilot study. These changes are explained in detail in the next sections.

**Table 2:** Changes made to the microinteraction ecological momentary assessment (μEMA) protocol or technology before the main study in response to the pilot participants’ feedback.

	Pilot study	Main study
Physical activity and sedentary behavior questions	Asked at random	Asked as sensor-triggered questions based on wrist-accelerometer activity
Location-based questions	Asked at random	Removed from μEMA and included as part of mobile phone EMA to obtain semantic location labels
μEMA question selection	At random	Using filter-based sampling algorithm
Person-level characteristics and validation questions	Not included in pilot study	Included in main study to assess validity and add variety to questions on μEMA days
Thank-you messages after answering a μEMA question	Messages selected at random from a bank of 10 messages	Messages selected sequentially from a bank of >250 messages
μEMA prompt wait time	15 seconds	Increased to 20 seconds
μEMA prompt vibration	11 seconds of progressively intense vibration pattern	6 seconds (2-second vibration with 1-second pause)

#### Changed μEMA Question-Selection Algorithm

In the pilot study, each μEMA prompt selected a question at random. As a result, the app had less control over preventing overrepresentation of questions within the day. On the basis of the interviews, we learned that participants found this repetitiveness of the questions monotonous. Thus, for the main study we developed a new question-selection algorithm that (1) guarantees that a given question is never asked consecutively and (2) ensures that a question is not answered more than a predetermined number of times.

#### Added Sensor-Triggered Questions

In the pilot study, we randomly sampled questions on PA, SB, and the participant’s current location. However, these questions also felt repetitive to the pilot participants. Thus, in the main study protocol, these questions were asked when the passive sensors (using accelerometer and GPS) detected a relevant event, gathering information *only* when it was contextually relevant.

#### Added μEMA Validation and Engagement Questions

We added 2 additional types of questions for μEMA. First, we included *validation questions* to check whether the participants are paying attention to the μEMA prompts. Second, we added questions related to the slowly changing characteristics of the participants; these questions add variety to the question pool while also gathering useful information. Finally, on the μEMA undo screen, we added more variety in the thank-you messages that participants receive after answering a μEMA prompt. As the main study would last for 12 months per participant, we deemed reducing burden and increasing engagement to be important considerations.

#### Optimized μEMA Interface

We increased the time duration that a μEMA question stays visible on the smartwatch face from 15 seconds to 20 seconds. To address participant concerns about the loud vibration, we changed the pattern from an 11-second-long vibration to a 6-second pattern of 2-second vibrations separated by 1-second pauses.

### Main TIME μEMA Substudy

The protocol described here is based on changes made to the μEMA app after the pilot study feedback.

### TIME Study Design

**Figure 3:**
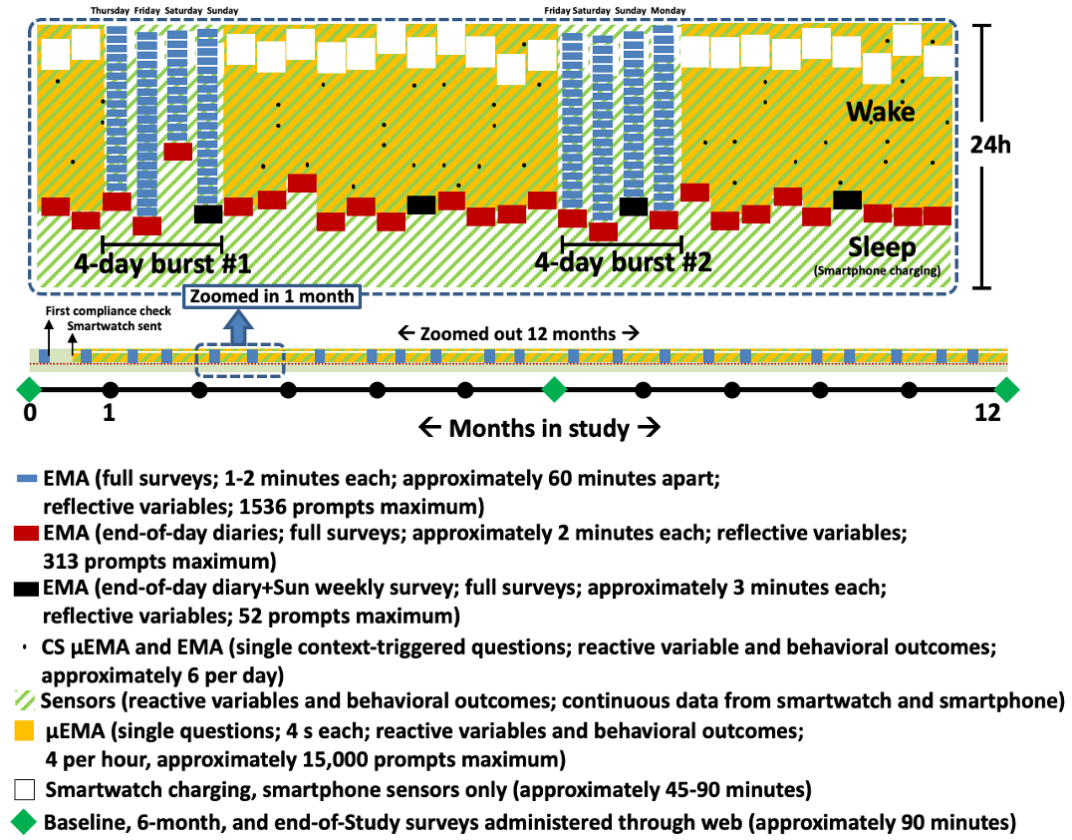
Temporal Influences on Movement & Exercise Study protocol with nested ecological momentary assessment (EMA) bursts and microinteraction EMA (μEMA) on nonburst days; CS: context-sensitive.

The TIME Study uses a nested design with multiple measurement bursts of smartphone-based EMA across a 12-month study period (Figure 3). Each measurement burst occurs over 4 days and always includes Saturday and Sunday. Measurement bursts occur every 2 weeks. The app ensures that there is at least a one-week gap between 2 consecutive burst periods. Thus, for a 12-month period (ie, 52 weeks), we expect up to 26 EMA burst periods (104 EMA burst days). The remaining nonburst days (or μEMA days) are reserved for μEMA prompting on the smartwatch; there can be up to 261 such days, with the number per participant dependent upon the receipt date of the smartwatch that is mailed only after the first successful burst period. The smartphone automatically sets the study schedule and controls the smartwatch so that μEMA questions are only prompted on the appropriate days and at the appropriate times. End-of-day surveys are prompted on all days, irrespective of measurement bursts. No μEMA prompts occur on the EMA burst days, and no other smartphone-based EMA prompts occur on μEMA days except for the end-of-day survey and sensor-triggered location surveys. Details of the smartphone-based EMA protocol are beyond the scope of this paper; they can be found in the main protocol paper (Wang, S, unpublished data, January 2022). Participants in the TIME Study are exposed to more interruptions from μEMA than from EMA, both within a day (4 μEMA prompts vs 1 EMA prompt per hour with possible additional reprompts) and across months (approximately 261 days for μEMA vs approximately 104 days for EMA for a year). Participants are also asked to complete baseline, 6-month, and 12-month surveys on the web using REDCap (Research Electronic Data Capture) [51]; the 6-month and 12-month surveys include questions about the perceived burden [52] of responding to μEMA and EMA questions. After the completion of the study, participants undergo a semistructured interview designed to gather additional information about their overall experiences answering questions on the devices. Participants who voluntarily withdraw from the study or who are asked to withdraw because of low EMA compliance are also asked to complete an exit survey to enable us to learn more about their experiences and reasons for withdrawal or poor compliance.

### Measures

Here we describe the self-report measures, question-selection algorithms, and simulation results to validate the filtering algorithm to select questions for μEMA.

### Self-report μEMA Measures

#### Core-Construct Questions

Core-construct questions measure different behaviors, momentary states, and contexts that cannot be objectively captured using sensors and are expected to change throughout the day. The questions are selected to capture reflective processes, reactive processes, and internal and external processes that can predict or explain behavior adoption and maintenance [49]. Example constructs include feelings of pain, stress, procrastination, happiness, and positive and negative affect. These questions are sampled from a question bank (based on a procedure defined later) consisting of 30 questions (Multimedia Appendix 1); they are presented with signal-contingent prompts based on the aforementioned schedule. Each day during waking hours, 90% (55/62) of the scheduled prompts are reserved for these core-construct questions. In the question bank of 30 core-construct questions, 6 (20%) measure reflective processes, 5 (17%) measure reactive processes, 6 (20%) measure external factors, and 13 (43%) measure internal factors.

#### Engagement and Validation Questions

In addition to the core-construct questions, 8% (5/62) of the scheduled prompts during waking hours are used to present questions about person-level characteristics. These are questions that gather data on attributes such as whether a participant owns a car, cares for a pet, self-describes as a morning person, or has other specific personality characteristics. The questions serve two purposes: gathering information that changes relatively slowly and breaking monotony (thus, perhaps helping with participant engagement) by *surprising* participants with approximately 4 novel questions a day. These questions follow the wording requirements of other μEMA questions (eg, *Own a pet?* with answers *Yes* or *No* or *Bike to work*? with answers *Frequently*, *Sometimes*, and *Rarely*). The system uses a question bank of >280 questions so that they will not repeat for approximately 3½ months (assuming 60 μEMA prompts per day).

The remaining 2% (1/62) of the waking-day prompts are used to present validation questions designed to assess whether participants are paying attention to the μEMA questions and answering them carefully (ie, not randomly tapping on the screen to dismiss a question without looking at it, carelessly answering prompts incorrectly, or accidently pressing buttons on the smartwatch without realizing it). The validation questions are like other μEMA questions—cognitively simple single questions with 2-3 answers. However, these questions always have an unambiguously correct answer to verify response accuracy. Validation questions include simple math problems (*1 + 3 = 5?*—*Yes* or *No*), trivial trivia questions (*Sun rises in the east?*—*Yes* or *No*), intentionally silly questions (*Do pigs fly?*—*Yes* or *No*), and simple attention-assessment questions (*Please press A:*—*A*, *B*, or *C*). To break the question monotony and enhance engagement, validation questions rarely repeat. Our item bank has >300 validation questions for an estimated 7½ months of question prompting without repetition.

#### PA and SB Questions

Sensor-triggered questions are prompted to measure PA and SB, which are indirectly, passively measured using sensors but may require self-report for validation. Participants are asked to report whether they are currently engaging in PA or SB based on their activity level as inferred from the smartwatch raw acceleration data (collected continuously at 50 Hz). In real time, we compute the area under the curve (AUC) of the high-passed raw accelerometer signal for 10-second epochs [53]. The AUC is then used to determine the prompting of sensor-triggered questions. If the AUC exceeds an empirically determined threshold in a continuous 10-minute window, a PA question is eligible to be asked. If the AUC falls below a threshold for a continuous 60-minute window, an SB question is eligible to be asked [53].

Sensor-triggered questions are prompted *only* when a specific sensor event of interest occurs that suggests contextual relevance. The questions are *Physically active [Δ] min ago?* and *Sedentary [Δ] min ago?*, with answer options *Yes*, *Sort of*, and *No*, where Δ is the time difference from the current time to the end of the activity plus 25% of the activity bout length (ie, 2½ minutes for a 10-minute PA bout or 15 minutes for a 60-minute sedentary bout). This additional 25% of bout length is used to ensure that the question is asked at approximately the moment in time when the behavior was likely taking place but not at the behavioral transition.

At each scheduled μEMA prompt time, if there is an eligible sensor-triggered question, the system will trigger the question only 50% of the time. This is done to avoid continuous and monotonous sensor-triggered prompting for prolonged sedentary and physical activities. When the sensor-triggered questions are eligible to be prompted, they take priority over the core-construct, validation, and engagement questions. For instance, if the next scheduled prompt has a core-construct question queued but the app has identified a PA event, then 50% of the time, that core-construct question is replaced by the PA confirmation question. If both PA and SB are identified, the question about the most recent event is presented.

### Question Selection for Prompting

A way to gather self-report data on a comprehensive set of behaviors using μEMA is to randomly select a question to ask the user—as was done in our pilot study. However, it may be useful to measure internal factors (eg, perceived stress, fatigue, and nervousness) more frequently within a day rather than external factors (eg, being with a friend). The app uses a strategic sampling algorithm to maximize the value of data collected about some constructs by limiting presentation of some questions, ensure that questions differ within an hour, and guarantee that each day includes questions from each of four broad construct categories (ie, internal and external factors and reflective and reactive processes).

### Selecting Day-Level Subset for Core-Construct Questions

Each μEMA day, from our bank of 30 μEMA questions of core constructs, the app selects 4 (13%) questions related to internal factors, 2 (7%) to external factors, 2 (7%) to reflective processes, and 2 (7%) to reactive processes. More internal factors than other categories are selected because they are expected to vary throughout the day [54]. Rather than selecting any question at random, this procedure ensures that at least one question measuring each of the different factors is presented each day, with a temporally dense presentation. Each question is expected to be presented ≥4 times in a day, allowing measurement of within-day changes.

### Filters Applied for Core-Construct Question Selection

#### Maximum Allowable Prompting per Question

Each μEMA question is assigned a maximum number of presentations per day (Multimedia Appendix 1). This ensures that some questions are not overrepresented in a day at the expense of others. For instance, questions capturing frequently changing constructs are assigned a higher limit (eg, *Feeling stressed?*) than questions that assess more stable constructs (eg, *Slept well yesterday?*). If the number of answers received for a question reaches the question’s limit, that question is no longer presented that day.

#### Minimum Time Gap for Question Repetition

Once a particular μEMA question has been answered, it will not be presented again until at least 60 minutes have elapsed. For instance, if the question *Feeling stressed?* is answered, it is not asked again for at least one hour. However, if the question is not answered, it could be presented at the next prompt, within the hour.

#### Filtering Algorithm When Selecting a μEMA Question

The app uses the following algorithm to select μEMA questions (Textbox 1). For each μEMA day (*μEMA_prompting_day*, line 1), the app first generates a day-level subset (*day_level_subset*, line 2) of 10 questions from the pool of 30 μEMA questions (*all_* μEMA *_questions*). Next, at each μEMA prompt, day-level questions that have reached their limit (*remove_maxed_out_questions*, line 4) or have been asked recently (*remove_questions_answered_within_one_hour*, line 5) are filtered. From the remaining list, a question is selected at random (*select_question_at_random*, line 6). If the question prompted is answered, the question’s prompt count is updated (*update_prompt_count*, line 8), as is the last-answered time (*update_answer_time*, line 9). If the question is not answered, then it is available for selection in the next scheduled μEMA prompt.

**Microinteraction ecological momentary assessment (μEMA) question-filtering algorithm at each prompt on nonburst days.**

**Algorithm used to select μEMA questions**

1. For each μEMA_prompting_day:
2. day_level_subset=subset (all_ μEMA _questions)
3. For each μEMA prompt:
4. filtered_questions=remove_maxed_out_questions (day_level_subset)
5. filtered_questions=remove_questions_answered_within_one_hour (filtered_questions)
6. question_to_ask=select_question_at_random (filtered_questions)
7. if question_to_ask is answered:
8. update_prompt_count (question_to_ask)
9. update_answer_time (question_to_ask)

### μEMA Question-Selection Simulation

To verify our filtering algorithm (Textbox 1), we simulated question sampling and compared the distributions of questions asked through random sampling (used in the pilot study) with our proposed protocol.

### Question-Sampling Algorithm Simulation

We simulated question selection for 365 days for 300 users, where 261 days were reserved for μEMA prompting, resulting in 78,300 total μEMA days. We assumed 8 hours of sleep and 16 hours of wake time per day, resulting in 15½ hours available for μEMA prompting. Targeting 4 prompts an hour results in 62 μEMA prompting opportunities. Of these, 90% (55/62) were available for core constructs, resulting in 55 slots for core-construct questions each day. We assumed that 75% (46/62) of the scheduled prompts would be answered (ie, compliance).

### Algorithm Simulation Results

Compared with random sampling (Multimedia Appendix 1), the filtering algorithm increased the frequency of presentation of core-construct questions in a day. For instance, when using random sampling, certain items such as sleep satisfaction are repeated at the expense of other variables of interest (eg, internal factors). Although with random sampling a question is prompted on more days (approximately 75% vs approximately 33%), the questions are repeated only twice (median frequency) on a given day. This presentation is insufficient to study within-day changes in slopes when we need at least three observations. The filtering algorithm ensures that on days when questions are presented, they are repeated 4 times (median frequency) a day. Later, we validate this algorithm against the real-world data from our main study participants (Multimedia Appendix 2).

### TIME Study Main Trial Participants and Recruitment

Participants were eligible for the TIME Study main trial if they (1) owned an Android smartphone running Android version ≥6.0 as their only personal mobile phone with no intention to switch to a non-Android smartphone, (2) did not wear a smartwatch already, (3) were aged 18-29 years and living in the United States, (4) were currently engaged in recommended levels of PA (or intended to within the next 12 months) [55], (5) spoke and read English, (6) resided in an area with Wi-Fi connectivity, (7) did not have any physical or cognitive limitations that prevented participation, and (8) were able wear a smartwatch and answer real-time smartphone and smartwatch surveys. All study procedures were approved by the institutional review board at the University of Southern California (USC; HS-18-00605). Participants were recruited using several strategies: (1) sending emails to individuals enrolled in the Happiness & Health Study, a USC longitudinal cohort study of young adults [56]; (2) posting flyers in the greater Los Angeles metropolitan area; (3) purchasing web and social media advertisements; (4) sending emails to addresses on file from other institutional review board–approved USC studies; and (5) contacting participants identified using ResearchMatch [57]. As of June 2021, 246 participants had been enrolled, among whom 81 (32.9%) had completed at least 6 months of data collection from their respective start dates (Table 3). We recruited 246 participants who received a smartwatch after the initial run-in period. This sample size was determined based on the objectives of the main TIME Study protocol (Wang, S, unpublished data, January 2022). The μEMA substudy was added (Figure 3) for exploratory purposes.

**Table 3:** Participant demographic survey for those who had completed 6 months as of June 2021 (N=81).

Demographics			Values					
Age (years), mean (SD)			21.7 (2.4)					
**Sex, n (%)**								
	Male					45 (55)		
	Female					36 (45)		
**Ethnicity, n (%)**								
	Non-Hispanic		51 (63)					
	Hispanic		30 (37)					
**Race^a^, n (%)**								
	White				41 (54)			
	Asian or Pacific Islander				35 (46)			
	Black				7 (9)			
	American Indian or Alaska Native				5 (7)			
**Education, n (%)**								
		High school		12 (15)				
		Some college		47 (58)				
		College graduate		22 (27)				
**Work status^b^, n (%)**								
		Employed					36 (45)	
		Out of work					16 (20)	
		Student					53 (66)	
		Unable to work					4 (5)	

^a^Participants could select >1 answer option from American Indian or Alaska Native, Hawaiian or Pacific Islander, Black or African American, White, Asian, Unknown, and Would prefer not to answer.

^b^Participants could select >1 answer option; for example, “Student” and “Employed.”

### Study Procedures

Because of COVID-19 restrictions on in-person recruitment for human subjects research, all participant recruitment and onboarding was conducted remotely. After screening, research staff individually met with each participant remotely (through Zoom) to obtain informed consent. Staff then guided the participants through the TIME app installation on their personal smartphones. Researchers then observed EMA burst compliance for the first 4-day EMA burst (ie, run-in period) during the next 2 weeks. If the compliance for the run-in period was <8 surveys per day on all days, the participants were withdrawn from the study. Otherwise, they could continue in the study and were mailed a smartwatch (Fossil Gen 4 or Gen 5 model). Once the participants received the smartwatch, a staff member scheduled a second remote orientation session with the participant to guide them through the TIME smartwatch setup. Expectations for smartwatch wear (including wearing it during sleep), charging it, and answering μEMA questions were explained. Participants were instructed that to remain compliant they should wear the smartwatch for 23 hours a day and charge it every day. They were also instructed to wear the smartwatch while sleeping. For μEMA prompting, participants were instructed as follows:

“You will be prompted on the smartwatch with single questions of the type Are you walking right now? Yes | Sort of | No. These questions will take only 2-4 seconds to respond to—just like checking time on the smartwatch. There may be up to six such question prompts in an hour, on average, and the frequency of question prompts may vary at different times of your waking day. All the smartwatch questions will be outside of the 4-day smartphone survey burst periods. Each watch prompt will wait for up to 20 seconds for you to respond; if the question is not answered within 20 seconds, the question may disappear from the watch. Unlike phone surveys, watch questions are not re-prompted.”

After the smartwatch orientation, participants could use the smartwatch as they deemed fit (if it did not interfere with TIME app functionality), while answering μEMA questions when they appeared. Participants receive US $20 per month for wearing the smartwatch for 23 hours on at least 24 days of the month. Participants could earn up to an additional US $80 per month for EMA burst compliance (Wang, S, unpublished data, January 2022). In addition, if at the end of the study, participants have >50% compliance with μEMA, they were able to keep the smartwatch as their personal device. However, no monthly or regular compensation was provided for μEMA compliance. Participants do not receive feedback on their μEMA compliance, and their withdrawal from the study does not depend on μEMA compliance.

### Response Behavior Measures

We measure compliance rate, completion rate, undo rate, and validation rate to characterize participant response behavior when answering μEMA questions.

#### Compliance Rate

Compliance rate is measured as the percentage of μEMA questions answered out of all the scheduled questions, including those not prompted because of the device being turned off or in DND mode.

Compliance rate (%) = #Questions answered/#Questions scheduled × 100

#### Completion Rate

Completion rate is measured as the percentage of μEMA questions answered out of all the delivered questions, excluding those not prompted because of the device being turned off or in DND mode.

Compliance rate (%) = #Questions answered/#Questions delivered × 100

#### Undo Rate

Undo rate is measured as the total number of μEMA questions answered when the *Undo?* option was selected after providing an initial answer.

Undo rate (%) = #Undo count/#Questions answered × 100

#### Validation Rate

Validation rate is measured as the percentage of μEMA validation questions answered correctly out of all the validation questions answered.

Validation rate (%) = #Validation correctly answered/#Validation answered × 100

## Results

### Compliance, Completion, and Validation Rates

Overall, the compliance rate (n=81 at ≥6 months) for μEMA was 67.7% (SD 13.7%) and the completion rate was 80.23% (SD 13.3%). The μEMA validation rate for the participants (n=81) was 92.9% (SD 23.7%)—indicating that they were paying attention to the μEMA question content and not carelessly tapping to dismiss them.

### Response Behavior

Table 4 presents a summary of the response behavior. We also verified the filter-based question-selection algorithm’s performance against the real-world data from 2% (2/81) of the study participants who completed 6 months in the study (Multimedia Appendix 2). We found that no core-construct question was asked more than its maximum allowable number of times. In addition, the median number of times a core-construct question was asked was consistent with our simulation results (Multimedia Appendix 1).

**Table 4:** Main study microinteraction ecological momentary assessment (μEMA) response behavior for participants completing 6 months as of June 2021 (N=81).

	Values
μEMA days, n	13,415
Expected μEMA questions, n	790,388
Delivered μEMA questions, n, %	662,397 (83.81)
Answered μEMA questions, n, %	535,430 (80.83)
Mean daily μEMA compliance rate, % (SD)	67.6 (24.4)
Mean daily μEMA completion rate, % (SD)	78.5 (22.2)
Mean participant μEMA compliance rate, % (SD)	67.4 (13.7)
Mean participant μEMA completion rate, % (SD)	80.2 (13.3)
Mean μEMA response time, seconds (SD)	4.8 (1.4)
Total μEMA undos (% of total μEMA questions answered)	22,202 (4.2)
Mean μEMA question validation rate, % (SD)	92.9 (23.7)

## Discussion

Our work with μEMA demonstrates that the technique may enable temporally dense ILD collection with manageable burden to support longitudinal studies or interventions, although some limitations of this work will require further investigation.

### Summary and Strengths

Traditional mobile phone–based EMA can lead to interruption and response burden. To prevent this burden, researchers often compromise on the temporal density of prompts (ie, by prompting less frequently) and reduce the number of questions or constructs being measured. Complementary forms of EMA are needed where both the researchers’ needs for comprehensive understanding of health behaviors and users’ concern for burden are taken into account. μEMA provides such an opportunity, where the quick microinteractions not only make it less burdensome for the users to answer questions on the smartwatch, but also enable data gathering at a higher temporal density.

The TIME μEMA substudy combines smartphone-based EMA with low-burden μEMA on a smartwatch to gather real-time self-report data from naturalistic settings using the personal smartphones of participants. Participants are encouraged to use their smartphone and the loaned smartwatch normally with only a few limitations (ie, avoiding apps that interfere with TIME app functionality, such as fitness trackers). Participants are incentivized to wear the smartwatch, but they can pause smartwatch survey prompting as needed. Participants are not directly compensated for high μEMA compliance, although they are told that they can keep the smartwatch if they have >50% μEMA compliance at the end of 1 year. EMA compliance, by contrast, is reinforced with an explicit and compliance-contingent reward provided at regular monthly intervals.

Our preliminary results from the pilot and ongoing TIME μEMA substudy show that despite a high interruption rate, μEMA could be sustainable for gathering self-report data longitudinally. Controlled filtering of μEMA questions enables measurement of a comprehensive set of behaviors repeatedly during waking hours. The validation questions make it possible to identify careless responding on both μEMA and smartphone-based EMA.

The TIME μEMA substudy can be used to assess the utility and sustainability of μEMA. Comparing response rates with the qualitative data from the exit interviews, for example, suggests that μEMA can be used to gather temporally dense self-report information. In addition, passively collected data on wrist motion (using accelerometers) and location (using GPS) may help explore contextual factors that affect μEMA compliance. The filtering-based μEMA question-selection algorithm may be used to capture diurnal patterns of different constructs during waking hours. These diurnal patterns could be used to explore (1) different clusters of individuals who have similar diurnal patterns for variables of interest [58] and (2) how similar, or different, diurnal patterns are that were captured using μEMA versus those captured using EMA. With 4 prompts per hour (or 70 prompts for an 18-hour wake period), μEMA may enable denser measurement than EMA. Combined with passive sensing, μEMA may support study of diurnal patterns, which might be compared with EMA at the end of the day.

Overall, the purpose of μEMA is not to replace traditional EMA but to complement it. The TIME Study’s μEMA substudy protocol provides an opportunity to explore how small amounts of information gathered at high frequency can help us learn about health behavior change and maintenance, even as constructs related to behavior change deviate by the hour. Beyond observation studies, TIME Study data could enable exploration of the viability of μEMA for longitudinal interventions (eg, just-in-time adaptive interventions), where self-report on many different behaviors may be required. For instance, when optimizing mobile health interventions as part of microrandomized trials [59], μEMA may provide a low-burden self-report interface that can gather information about behaviors that sensors cannot measure yet (eg, fatigue, procrastination, and pain), especially when such measurements are needed in close time intervals (eg, within an hour) and across changing contexts.

### Limitations and Opportunities

There will be several limitations of the TIME μEMA substudy that will provide opportunities for future research. Findings about the μEMA methodology may not generalize to other population age groups because the main protocol is designed to study health behavior adoption and maintenance in young adults (age 18-29 years). There has been an increased interest in exploring the acceptance of wearable technologies in older adults [60-62], youth [63], and children [64] and more research is needed to explore the acceptance of μEMA as a data collection method for both longitudinal observational and intervention studies with these demographic groups. As μEMA is deployed on a wearable smartwatch, our data collection excludes participants who may not be allowed to wear, or be comfortable wearing, the smartwatch during work hours; in some professions, answering μEMA or EMA surveys may not be appropriate (eg, professions that require being in intensive care units, using cleanrooms, extended driving, operating heavy machinery, and working in construction).

We had to restrict our data collection to Android users because our research app used advanced sensor capabilities not programmatically available on iOS devices; thus, the TIME μEMA substudy excludes iOS users. Nevertheless, we observed more demographic diversity in the Android users who participated in our screening surveys (of the 746 participants, 393, 52.7%, were women; 372, 49.9%, were White; 216, 28.9%, were Asian; 119, 15.9%, were Black; and 184, 24.6%, were Hispanic; 630, 84.5%, engage in PA) versus the iOS users (of the 395 participants, 296, 75.1%, were women; 177, 44.9%, were White; 99, 25.1%, were Asian; 71, 17.8%, were Black, and 130, 32.9% were Hispanic; 365, 92.4%, engage in PA). We have no reason to believe that the platform itself would affect μEMA response patterns.

Although μEMA may keep burden more manageable than EMA despite intensive prompting, each question provides a limited answer set (*Yes*, *Sort of,* and *No*) that may be less sensitive to construct variance than mobile phone–based EMAs with more response options. Yet, with a median of ≥4 measurements per construct within a day (Multimedia Appendix 1), μEMA provides opportunities to use mixed effects location scale models to study changes in variances and slopes of different constructs [65,66]. This also provides opportunities to explore approaches beyond multilevel models, for instance, using dynamic Bayesian networks [67,68] or temporal networks [69,70] to build individual-specific models.

Unlike EMA, where multiple questions are asked back to back, thus allowing for the simultaneous comparison of different behaviors, μEMA presents only 1 question at a time to ensure that each prompt can be responded to as a microinteraction. Therefore, different construct measurements are spread throughout waking hours at different times. Although this approach does not allow for testing concurrent associations among constructs, there are alternative ways of exploring temporally lagged associations of different μEMA measures (eg, Granger Causality [71]).

### Conclusions

The TIME μEMA substudy provides an opportunity to explore a new method of collecting temporally dense self-report data. Although EMA provides information on temporal dynamics of behavior, the burden of accessing the mobile phone, unlocking it, and then answering multiple complex questions compromises engagement. In this formative research, we explored the feasibility of using μEMA to measure multiple constructs in an ILD study. When deploying μEMA, each prompt is a cognitively simple single question that can be answered with a quick glanceable microinteraction with an always-accessible smartwatch. Because of this simplicity, μEMA permits a higher interruption rate than EMA without a corresponding increase in perceived burden. The TIME Study is the first ILD study to deploy μEMA for an entire year; thus, it could provide insights on methodological properties of μEMA—especially the ability to sustain participant engagement. Data from the study could be used to study what influences μEMA compliance, what can be learned about an individual’s behavior using μEMA, and how μEMA might be used along with EMA and passive sensors to richly characterize human behavior, especially how it may change throughout the day in response to rapidly changing contexts.

## Appendix

Multimedia Appendix 1 Random versus filter-based question sampling.

Multimedia Appendix 2 Filter-based sampling simulation versus main study participant results.

## Abbreviations

AUC: area under the curve
DND: do not disturb
EMA: ecological momentary assessment
ILD: intensive longitudinal data
PA: physical activity
REDCap: Research Electronic Data Capture
SB: sedentary behavior
TIME: Temporal Influences on Movement & Exercise
UI: user interface
μEMA: microinteraction ecological momentary assessment
