# Supplementary material for: Intensive Longitudinal Data Collection Using Microinteraction Ecological Momentary Assessment: Pilot and Preliminary Results
Source: JMIR Form Res. 2022 Feb 9;6(2):e32772. doi: 10.2196/32772 (PMC8867293; doi:10.2196/32772)
Supplement: Multimedia Appendix 1 [file formative_v6i2e32772_app1.docx]

## Multimedia Appendix 1: Random vs Filter-based Question Sampling

In this table, we present the results of simulating prompts for a year (assuming 261 μEMA days) for a hypothetical participant to verify the filter-based approach of question selection. We assume a 75% compliance rate and 8 h of sleep duration. With those assumptions and random sampling, each question would be asked on ~75% of days, and typically between 1-2 times. The filter-based approach ensures daily question representation from four categories of external factors (EF), internal factors (IF), reflective processes (RefP), and reactive processes (ReaP) that might influence health behavior change and maintenance in young adults, while also guaranteeing that questions are not asked too often, and most questions are asked with appropriate within-day temporal density. All the μEMA questions have ‘Yes/Sort of/No’ answer options.

**Table 6. μEMA core-construct questions, grouped by type, and expected number of responses each day using the filter-based sampling approach. For each question, the table shows the maximum allowable prompts answered per day (Max allowed/Day), the percentage of days of the year on which the question will be used (% Days), the total number of days on which the question will be used (~Total days), and the minimum and median times on a day when the question is presented that it is expected to be answered (Min/Day and Med/Day).**

|  | | | Expected answered observations per participant from filter-based sampling | | | |
| --- | --- | --- | --- | --- | --- | --- |
| μEMA Question | Type | Max allowed/day | % days in the year | ~Total days | Min/ day | Med/ day |
| With family member(s)? | EF | 6 | 34.16 | 92 | 1 | 4 |
| Workload increasing? | EF | 6 | 33.4 | 90 | 1 | 4 |
| Alone right now? | EF | 6 | 33.07 | 89 | 1 | 4 |
| With friend(s)? | EF | 6 | 33.1 | 89 | 1 | 4 |
| Feeling pain/sick? | EF | 10 | 32.96 | 89 | 1 | 4 |
| Juggling several tasks? | EF | 10 | 32.73 | 88 | 1 | 4 |
| Feeling fatigued? | IF | 10 | 31.8 | 86 | 1 | 4 |
| Feeling happy? | IF | 10 | 31.67 | 86 | 1 | 4 |
| Feeling sad? | IF | 10 | 31.36 | 85 | 1 | 4 |
| Feeling frustrated? | IF | 10 | 31.19 | 84 | 1 | 4 |
| Feeling nervous? | IF | 10 | 31.24 | 84 | 1 | 4 |
| Feeling energetic? | IF | 10 | 29.77 | 83 | 1 | 4 |
| Slept well yesterday? | IF | 1 | 30.65 | 83 | 1 | 1 |
| Feeling hungry? | IF | 6 | 30.23 | 82 | 1 | 4 |
| Feeling tense? | IF | 10 | 30.42 | 82 | 1 | 4 |
| Feeling tired? | IF | 10 | 30.52 | 82 | 1 | 4 |
| Feeling excited? | IF | 10 | 30.14 | 81 | 1 | 4 |
| Feeling relaxed? | IF | 10 | 30.14 | 81 | 1 | 4 |
| Feeling stressed? | IF | 10 | 29.62 | 80 | 1 | 4 |
| Feeling focused? | ReaP | 10 | 40.34 | 109 | 1 | 4 |
| On usual routine? | ReaP | 10 | 39.91 | 108 | 1 | 4 |
| Feel like eating healthy? | ReaP | 6 | 39.53 | 107 | 1 | 4 |
| Feel like exercising? | ReaP | 6 | 39.36 | 106 | 1 | 4 |
| Feel like sitting less? | ReaP | 6 | 33.08 | 89 | 1 | 4 |
| Feeling in control? | RefP | 10 | 34.02 | 92 | 1 | 4 |
| Intend to eat healthy? | RefP | 4 | 33.18 | 90 | 1 | 4 |
| Intend to exercise? | RefP | 4 | 33.24 | 90 | 1 | 4 |
| Intend to sit less? | RefP | 4 | 33.08 | 89 | 1 | 4 |
| Procrastinating? | RefP | 10 | 32.82 | 89 | 1 | 4 |
| Feeling productive? | RefP | 10 | 33.04 | 89 | 1 | 4 |

^*IF: Internal factors, EF: External factors, ReaP: Reactive processes, RefP: Reflective processes^
