# Supplementary material for: Intensive Longitudinal Data Collection Using Microinteraction Ecological Momentary Assessment: Pilot and Preliminary Results
Source: JMIR Form Res. 2022 Feb 9;6(2):e32772. doi: 10.2196/32772 (PMC8867293; doi:10.2196/32772)
Supplement: Multimedia Appendix 2 [file formative_v6i2e32772_app2.docx]

## Multimedia Appendix 2: Filter-based Sampling Simulation vs Main Study Participant Results

Here, we present the μEMA answered question distribution from two active main study participants (P1 and P2) who were in the study for 153 and 150 μEMA days with a compliance of 77% and 70% respectively. The participants answered 5,511 and 5,754 core-construct μEMA questions, respectively. The filter-based prompting strategy performed as expected.

**Table 7. Distribution of μEMA core-construct question type responses for two study participants who completed 153 and 150 days of μEMA data collection. The table shows the percentage and number of days each question was answered at least once (% Days and Total days), the minimum, median, and maximum times a day answers were obtained (Min/Day, Med/Day, Max/Day), and the total number of answers received (Total answered).**

|  | | | P1 (Compliance 77%, Completion 93%, days in study = 153) | | | | | | P2 (Compliance 70%, Completion 90%, days in study = 150) | | | | | |
| --- | --- | --- | --- | --- | --- | --- | --- | --- | --- | --- | --- | --- | --- | --- |
| μEMA Question | Type | | % Days | Total days | Min/ Day | Med/ Day | Max/ Day | Total answered | % Days | Total days | Min/ Day | Med/ Day | Max/ Day | Total answered |
| With family member (s)? | | EF | 29.71 | 41 | 1 | 4 | 6 | 159 | 31.21 | 49 | 1 | 4 | 5 | 190 |
| Workload increasing? | | EF | 30.43 | 48 | 1 | 4 | 6 | 164 | 27.39 | 43 | 1 | 4 | 6 | 169 |
| Alone right now? | | EF | 34.78 | 48 | 1 | 4 | 6 | 196 | 26.11 | 41 | 1 | 4 | 6 | 159 |
| With friend(s)? | | EF | 30.43 | 42 | 1 | 4 | 6 | 170 | 29.94 | 47 | 1 | 4 | 6 | 183 |
| Feeling pain/sick? | | EF | 30.43 | 42 | 1 | 5 | 9 | 201 | 33.12 | 52 | 1 | 4 | 7 | 196 |
| Juggling several tasks? | | EF | 36.96 | 51 | 1 | 5 | 9 | 236 | 41.4 | 65 | 1 | 4 | 9 | 270 |
| Feeling fatigued? | | IF | 35.51 | 49 | 1 | 5 | 8 | 211 | 29.3 | 46 | 1 | 3 | 6 | 179 |
| Feeling happy? | | IF | 24.64 | 34 | 1 | 4 | 7 | 138 | 29.94 | 47 | 1 | 4 | 6 | 181 |
| Feeling sad? | | IF | 23.91 | 33 | 1 | 4 | 8 | 149 | 30.57 | 48 | 2 | 4 | 8 | 191 |
| Feeling frustrated? | | IF | 24.64 | 34 | 1 | 4 | 7 | 139 | 27.39 | 43 | 1 | 4 | 6 | 168 |
| Feeling nervous? | | IF | 31.16 | 43 | 1 | 3 | 7 | 167 | 33.12 | 52 | 1 | 4 | 9 | 219 |
| Feeling energetic? | | IF | 25.36 | 35 | 1 | 5 | 7 | 149 | 30.57 | 48 | 1 | 4 | 7 | 198 |
| Slept well yesterday? | | IF | 23.91 | 33 | 1 | 1 | 1 | 33 | 32.48 | 51 | 1 | 1 | 1 | 51 |
| Feeling hungry? | | IF | 34.78 | 48 | 1 | 4 | 6 | 183 | 35.67 | 56 | 1 | 5 | 6 | 238 |
| Feeling tense? | | IF | 34.06 | 47 | 1 | 4 | 8 | 194 | 26.11 | 41 | 1 | 3 | 6 | 155 |
| Feeling tired? | | IF | 40.58 | 56 | 2 | 4 | 9 | 242 | 30.57 | 48 | 1 | 4 | 7 | 191 |
| Feeling excited? | | IF | 25.36 | 35 | 1 | 5 | 8 | 171 | 27.39 | 43 | 1 | 4 | 7 | 184 |
| Feeling relaxed? | | IF | 35.51 | 49 | 1 | 4 | 8 | 199 | 34.39 | 54 | 1 | 4 | 6 | 204 |
| Feeling stressed? | | IF | 34.06 | 47 | 1 | 5 | 8 | 228 | 23.57 | 37 | 1 | 4 | 7 | 134 |
| Feeling focused? | | ReaP | 35.51 | 49 | 1 | 4 | 7 | 207 | 36.94 | 58 | 2 | 4 | 9 | 248 |
| On usual routine? | | ReaP | 44.2 | 61 | 1 | 4 | 10 | 281 | 43.31 | 68 | 1 | 4 | 8 | 267 |
| Feel like eating healthy? | | ReaP | 45.65 | 63 | 1 | 4 | 6 | 263 | 43.95 | 69 | 1 | 4 | 6 | 282 |
| Feel like exercising? | | ReaP | 36.23 | 50 | 1 | 4 | 6 | 202 | 35.03 | 55 | 1 | 3 | 6 | 206 |
| Feel like sitting less? | | ReaP | 36.96 | 51 | 1 | 4 | 6 | 215 | 35.03 | 55 | 1 | 4 | 6 | 222 |
| Feeling in control? | | RefP | 38.41 | 53 | 1 | 4 | 8 | 197 | 31.85 | 50 | 1 | 5 | 7 | 200 |
| Intend to eat healthy? | | RefP | 28.86 | 39 | 1 | 4 | 4 | 136 | 34.39 | 54 | 1 | 3 | 4 | 176 |
| Intend to exercise? | | RefP | 27.54 | 38 | 1 | 4 | 4 | 123 | 32.48 | 51 | 1 | 3 | 4 | 155 |
| Intend to sit less? | | RefP | 34.78 | 48 | 1 | 4 | 4 | 165 | 28.03 | 44 | 2 | 4 | 4 | 151 |
| Procrastinating? | | RefP | 31.16 | 43 | 1 | 5 | 8 | 196 | 31.21 | 49 | 1 | 4 | 6 | 185 |
| Feeling productive? | | RefP | 35.51 | 49 | 1 | 4 | 8 | 198 | 34.39 | 54 | 1 | 4 | 7 | 202 |

^*IF: Internal factors, EF: External factors, ReaP: Reactive processes, RefP: Reflective processes^
